# Supplementary material for: Risk of COVID-19 death in adults who received booster COVID-19 vaccinations in England
Source: Nat Commun. 2024 Jan 16;15:398. doi: 10.1038/s41467-023-44276-x (PMC10791661; doi:10.1038/s41467-023-44276-x)
Supplement: Supplementary file 1 — Supplementary Information [file 41467_2023_44276_MOESM1_ESM.pdf]

Supplementary

Supplementary Figure 1

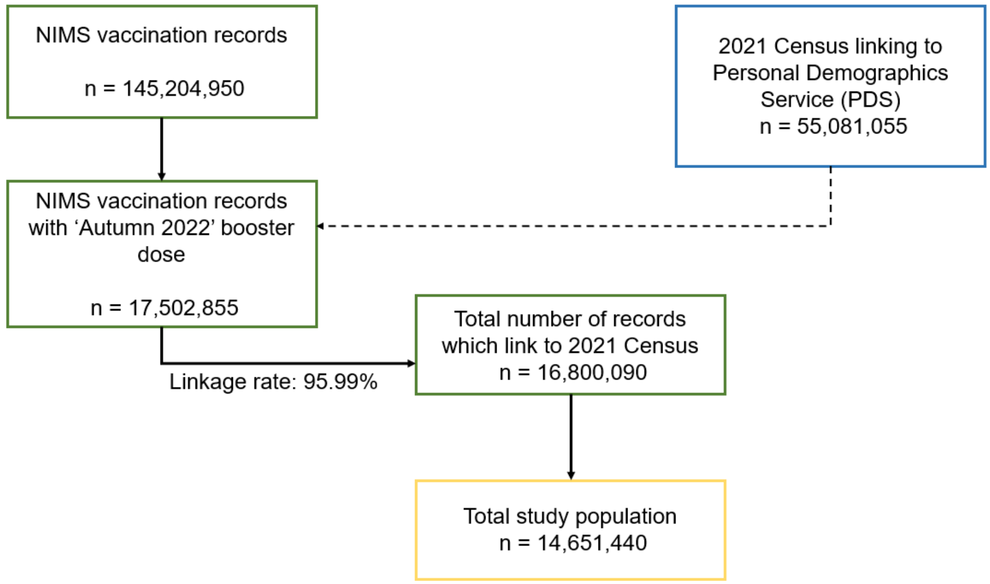

Population diagram: *Sample flow diagram detailing derivation of study population with counts (n) shown rounded to the nearest 5.*

***Supplementary Table 1** – Population sample flow*

**Stage**	**Total population**
Total 2021 Census linking to Personal Demographics Service (PDS)	55,081,055
Total number of NIMS records	145,204,950
Total number of NIMS records with autumn 2022 dose	17,502,855
Total number of NIMS records with autumn 2022 dose which link to 2021 Census	16,800,090
Total population who were usual residents at time of 2021 Census	16,777,280
Total population aged 50 to 100 years of age on vaccination date and alive on 1 September 2022	14,657,455
Total people with positive time at risk (time between 14 days after vaccination and the earliest of end of study (11 April 2023) date or date of death)	14,651,440

Supplementary Table 2 – Z-statistics estimates for health predictors from bootstrapped estimates

**Category**	**Z-statistic**
Asthma	1.32
Atrial Fibrillation	-0.32
Cancer of Blood or Bone Marrow	5.96
Chronic Kidney Disease	3.06
Congenital Heart Problem	-0.93
Chronic obstructive pulmonary disease (COPD)	0.54
Coronary Heart Disease	1.07
Cystic fibrosis *	3.47
Dementia	-7.15
Diabetes: Type 1	0.03
Diabetes: Type 2	-1.02
Epilepsy	-0.69
Heart Failure	0.79
Immunosuppressed	1.26
Learning Disability or Down Syndrome	1.15
Prescribed leukotriene	0.98
Liver Cirrhosis	-1.86
Lung or Oral Cancer	-0.04
Motor neurone disease /Multiple sclerosis/Myasthenia/Huntington’s/Chorea	0.23
Parkinson’s Disease	0.36
Peripheral Vascular Disease	-1.58
Prior Fracture ***	-1.16
Pulmonary Hypertension or Fibrosis	2.45
Rheumatoid Arthritis or Systemic lupus erythematosus (SLE)	2.77
Schizophrenia	0.13
Severe mental illness	0.21
Stroke **	-0.07
Thrombosis or pulmonary embolus	0.34

* - Cystic fibrosis, bronchiectasis or alveolitis, ** - Stroke or transient ischaemic attack (TIA), *** - Prior fracture of hip, wrist, spine, or humerus

Supplementary Table 3 – Health conditions associated with COVID-19 and non-COVID-19 death from model estimates not adjusted for all other health conditions

**Category**	**HR for COVID-19 death (95% CI)**	**HR for non-COVID-19 death (95% CI)**
Asthma	1.21 (1.1 - 1.33)	1.07 (1.05 - 1.09)
Atrial Fibrillation	1.6 (1.5 - 1.71)	1.58 (1.56 - 1.6)
Cancer of Blood or Bone Marrow	3.58 (3.14 - 4.07)	1.9 (1.83 - 1.97)
Chronic Kidney Disease	1.55 (1.46 - 1.65)	1.38 (1.36 - 1.4)
Congenital Heart Problem	1.37 (0.78 - 2.41)	1.59 (1.43 - 1.78)
Chronic obstructive pulmonary disease (COPD)	2.51 (2.35 - 2.69)	2.3 (2.27 - 2.34)
Coronary Heart Disease	1.43 (1.32 - 1.53)	1.33 (1.31 - 1.35)
Cystic fibrosis *	2.5 (2.17 - 2.88)	1.65 (1.59 - 1.72)
Dementia	2.83 (2.63 - 3.04)	3.66 (3.61 - 3.71)
Diabetes: Type 1	1.66 (1.48 - 1.85)	1.68 (1.65 - 1.72)
Diabetes: Type 2	1.46 (1.37 - 1.55)	1.51 (1.49 - 1.53)
Epilepsy	1.81 (1.41 - 2.33)	2.05 (1.95 - 2.15)
Heart Failure	2.34 (2.18 - 2.52)	2.19 (2.15 - 2.23)
Immunosuppressed	2.62 (2.34 - 2.93)	1.88 (1.83 - 1.93)
Learning Disability or Down Syndrome	5.49 (4 - 7.53)	4.72 (4.42 - 5.04)
Prescribed leukotriene	1.79 (1.69 - 1.89)	1.61 (1.59 - 1.63)
Liver Cirrhosis	3.52 (2.6 - 4.77)	4.48 (4.23 - 4.73)
Lung or Oral Cancer	3.82 (3.03 - 4.8)	3.66 (3.48 - 3.84)
Motor neurone disease /Multiple sclerosis/Myasthenia/Huntington's/Chorea	3.44 (2.14 - 5.54)	3.15 (2.84 - 3.5)
Parkinson's Disease	3.08 (2.64 - 3.59)	3.18 (3.08 - 3.29)
Peripheral Vascular Disease	1.85 (1.61 - 2.13)	2 (1.94 - 2.06)
Prior Fracture ***	1.37 (0.95 - 1.97)	1.62 (1.51 - 1.74)
Pulmonary Hypertension or Fibrosis	4.09 (3.46 - 4.83)	2.9 (2.78 - 3.03)
Rheumatoid Arthritis or Systemic lupus erythematosus (SLE)	2.26 (1.95 - 2.62)	1.52 (1.47 - 1.58)
Schizophrenia	2.5 (1.89 - 3.31)	2.45 (2.31 - 2.6)
Severe mental illness	1.67 (1.52 - 1.83)	1.66 (1.63 - 1.69)
Stroke **	1.56 (1.43 - 1.7)	1.6 (1.58 - 1.63)
Thrombosis or pulmonary embolus	2.53 (0.82 - 7.85)	1.97 (1.49 - 2.59)

* - Cystic fibrosis, bronchiectasis or alveolitis, ** - Stroke or transient ischaemic attack (TIA), *** - Prior fracture of hip, wrist, spine, or humerus
