# Supplementary material for: Risk of COVID-19 death in adults who received booster COVID-19 vaccinations in England
Source: Nat Commun. 2024 Jan 16;15:398. doi: 10.1038/s41467-023-44276-x (PMC10791661; doi:10.1038/s41467-023-44276-x)
Supplement: Supplementary file 3 — Description of Additional Supplementary Files [file 41467_2023_44276_MOESM3_ESM.pdf]

<u>Description of Additional Supplementary files</u>

Supplementary Data 1: Characteristics of the study population, predictor groupings and deaths

* - Cystic fibrosis, bronchiectasis or alveolitis, ** - Stroke or transient ischaemic attack (TIA), *** - Prior fracture of hip, wrist, spine or humerus

Supplementary Data 2: Sociodemographic characteristics and health conditions associated with COVID-19 and non-COVID-19 death

Hazard ratios (HRs) were calculated with a Cox regression model which was adjusted for: ***age****,* ***sex,*** *and calendar time (Model 1), plus* ***ethnic group*** *(Model 2), and* ***BMI*** *(Model 3), and* ***disability*** *(Model 4) and* ***health conditions*** *(Model 5).*

* - Cystic fibrosis, bronchiectasis or alveolitis, ** - Stroke or transient ischaemic attack (TIA), *** - Prior fracture of hip, wrist, spine or humerus
