# Supplementary material for: Risk of COVID-19 death in adults who received booster COVID-19 vaccinations in England
Source: Nat Commun. 2024 Jan 16;15:398. doi: 10.1038/s41467-023-44276-x (PMC10791661; doi:10.1038/s41467-023-44276-x)
Supplement: Supplementary file 6 — Reporting Summary [file 41467_2023_44276_MOESM6_ESM.pdf]

nature portfolio

Corresponding author(s): Isobel Ward

Last updated by author(s): 2023/11/06

Reporting Summary

Nature Portfolio wishes to improve the reproducibility of the work that we publish. This form provides structure for consistency and transparency in reporting. For further information on Nature Portfolio policies, see our Editorial Policies and the Editorial Policy Checklist.

Statistics

For all statistical analyses, confirm that the following items are present in the figure legend, table legend, main text, or Methods section.

n/a	Confirmed	
☐	☒	The exact sample size (*n*) for each experimental group/condition, given as a discrete number and unit of measurement
☐	☒	A statement on whether measurements were taken from distinct samples or whether the same sample was measured repeatedly
☒	☐	The statistical test(s) used AND whether they are one- or two-sided<br>*Only common tests should be described solely by name; describe more complex techniques in the Methods section.*
☐	☒	A description of all covariates tested
☐	☒	A description of any assumptions or corrections, such as tests of normality and adjustment for multiple comparisons
☐	☒	A full description of the statistical parameters including central tendency (e.g. means) or other basic estimates (e.g. regression coefficient) AND variation (e.g. standard deviation) or associated estimates of uncertainty (e.g. confidence intervals)
☒	☐	For null hypothesis testing, the test statistic (e.g. *F*, *t*, *r*) with confidence intervals, effect sizes, degrees of freedom and *P* value noted<br>*Give P values as exact values whenever suitable.*
☒	☐	For Bayesian analysis, information on the choice of priors and Markov chain Monte Carlo settings
☒	☐	For hierarchical and complex designs, identification of the appropriate level for tests and full reporting of outcomes
☒	☐	Estimates of effect sizes (e.g. Cohen's *d*, Pearson's *r*), indicating how they were calculated

Our web collection on statistics for biologists contains articles on many of the points above.

Software and code

Policy information about availability of computer code

Data collection	Data linkage and preparation was conducted using R 3.5, Python version 3.6 and Spark version 2.4
Data analysis	All analyses were conducted using R 3.5.

For manuscripts utilizing custom algorithms or software that are central to the research but not yet described in published literature, software must be made available to editors and reviewers. We strongly encourage code deposition in a community repository (e.g. GitHub). See the Nature Portfolio guidelines for submitting code & software for further information.

Data

Policy information about availability of data

All manuscripts must include a data availability statement. This statement should provide the following information, where applicable:

- Accession codes, unique identifiers, or web links for publicly available datasets
- A description of any restrictions on data availability
- For clinical datasets or third party data, please ensure that the statement adheres to our policy

The source data used in this study is subject to controlled access due to its sensitive nature. All statistical data used in this study are available from the Office for National Statistics website.

Research involving human participants, their data, or biological material

Policy information about studies with human participants or human data. See also policy information about sex, gender (identity/presentation), and sexual orientation and race, ethnicity and racism.

Reporting on sex and gender	No human research participants were involved in the research. Only administrative data sources were used. Where sex is reported, it refers to self-reported sex as recorded in the 2011 Census.
Reporting on race, ethnicity, or other socially relevant groupings	No human research participants were involved in the research.
Population characteristics	Population characteristics (sex, ethnic group, age, disability status) were self reported from Census 2011. A full description of the study population characteristics is reported in Table 1.
Recruitment	No human research participants were involved in the research.
Ethics oversight	This study was approved by the National Statisticians Ethics Board and was conducted in accordance with the Declaration of Helsinki. Administrative data was used, therefore all individuals whose data were collected in line with the data collection policies for each data source were included.

Note that full information on the approval of the study protocol must also be provided in the manuscript.

Field-specific reporting

Please select the one below that is the best fit for your research. If you are not sure, read the appropriate sections before making your selection.

☐ Life sciences ☒ Behavioural & social sciences ☐ Ecological, evolutionary & environmental sciences

For a reference copy of the document with all sections, see nature.com/documents/nr-reporting-summary-flat.pdf

Behavioural & social sciences study design

All studies must disclose on these points even when the disclosure is negative.

Study description	National retrospective cohort study, es, qualitative data
Research sample	Adults aged 50-100-years in England vaccinated with a booster in autumn 2022 and enumerated in Census 2021. Our total population was 14,644,570 people; there were 6,800 COVID-19 deaths and 150,075 non-COVID-19 deaths. This cohort was selected as it consisted of the whole population in England who had been vaccinated with a COVID-19 booster as part of the autumn 2022 campaign and recorded in NIMS.
Sampling strategy	No sampling was needed. The full dataset was used for analysis. No qualitative data was used.
Data collection	Administrative data was used. Therefore the data was recorded in response to an event occurring e.g. death/vaccination and not necessarily for the purposes of research e.g. use of mortality data from death registration certificates and use of vaccination data from the National Immunisation Management System (NIMS) used to record vaccinations in the population to aid with the vaccinations roll-out. The researchers were not involved in the collection of any of the data used in the analysis.
Timing	This study was a retrospective cohort study that included individuals vaccinated with a COVID-19 booster dose in England after September 1, 2022, were enumerated in Census 2021, linked to the PDS, and were aged 50-100-years of age on the date of booster administration. An autumn booster dose was defined as a booster dose administered on or after September 1, 2022, and at least 84 days since the last clinically acceptable dose, and individuals must have had at least two doses prior to the booster (Supplementary Figure 1). Individuals were followed from 14-days after the autumn dose booster vaccination date until April 11, 2023. Time at risk started 14 days after booster vaccination and ended at time of death (either COVID-19 or other), or end of study (April 11, 2023).
Data exclusions	From the 17,502,855 persons with NIMS autumn booster vaccination records, 16,800,090 people could be linked to Census 2021. Only usual residents were included (n=16,777,280), and adults aged 50 to 100 years of age (n=14,657,455) and finally those who survived till the start of the follow up period 14 days after vaccination. Our total population included 14,651,440 people.
Non-participation	Administrative data was used, therefore all individuals whose data were collected in line with the data collection policies for each data source were included.
Randomization	No randomization was applied. Time-varying factors were controlled for by the inclusion of calendar time as a covariate.

Reporting for specific materials, systems and methods

We require information from authors about some types of materials, experimental systems and methods used in many studies. Here, indicate whether each material, system or method listed is relevant to your study. If you are not sure if a list item applies to your research, read the appropriate section before selecting a response.

Materials & experimental systems

n/a	Involved in the study
☒	☐ Antibodies
☒	☐ Eukaryotic cell lines
☒	☐ Palaeontology and archaeology
☒	☐ Animals and other organisms
☒	☐ Clinical data
☒	☐ Dual use research of concern
☒	☐ Plants

Methods

n/a	Involved in the study
☒	☐ ChIP-seq
☒	☐ Flow cytometry
☒	☐ MRI-based neuroimaging

Plants

Seed stocks	*Report on the source of all seed stocks or other plant material used. If applicable, state the seed stock centre and catalogue number. If plant specimens were collected from the field, describe the collection location, date and sampling procedures.*
Novel plant genotypes	*Describe the methods by which all novel plant genotypes were produced. This includes those generated by transgenic approaches, gene editing, chemical/radiation-based mutagenesis and hybridization. For transgenic lines, describe the transformation method, the number of independent lines analyzed and the generation upon which experiments were performed. For gene-edited lines, describe the editor used, the endogenous sequence targeted for editing, the targeting guide RNA sequence (if applicable) and how the editor was applied.*
Authentication	*Describe any authentication procedures for each seed stock used or novel genotype generated. Describe any experiments used to assess the effect of a mutation and, where applicable, how potential secondary effects (e.g. second site T-DNA insertions, mosiacism, off-target gene editing) were examined.*
